# Transcriptional activity around bacterial cell death reveals molecular biomarkers for cell viability

**DOI:** 10.1186/1471-2164-9-590

**Published:** 2008-12-06

**Authors:** Remco Kort, Bart J Keijser, Martien PM Caspers, Frank H Schuren, Roy Montijn

**Affiliations:** 1 TNO Quality of Life, Business Unit Food and Biotechnology Innovations, Microbial Genomics Group, Utrechtseweg 48, PO Box 360, 3700AJ Zeist, The Netherlands

## Abstract

**Background:**

In bacteriology, the ability to grow in selective media and to form colonies on nutrient agar plates is routinely used as a retrospective criterion for the detection of living bacteria. However, the utilization of indicators for bacterial viability-such as the presence of specific transcripts or membrane integrity-would overcome bias introduced by cultivation and reduces the time span of analysis from initiation to read out. Therefore, we investigated the correlation between transcriptional activity, membrane integrity and cultivation-based viability in the Gram-positive model bacterium *Bacillus subtilis*.

**Results:**

We present microbiological, cytological and molecular analyses of the physiological response to lethal heat stress under accurately defined conditions through systematic sampling of bacteria from a single culture exposed to gradually increasing temperatures. We identified a coherent transcriptional program including known heat shock responses as well as the rapid expression of a small number of sporulation and competence genes, the latter only known to be active in the stationary growth phase.

**Conclusion:**

The observed coordinated gene expression continued even after cell death, in other words after all bacteria permanently lost their ability to reproduce. Transcription of a very limited number of genes correlated with cell viability under the applied killing regime. The transcripts of the expressed genes in living bacteria – but silent in dead bacteria-include those of essential genes encoding chaperones of the protein folding machinery and can serve as molecular biomarkers for bacterial cell viability.

## Background

Ever since the pioneering work by Louis Pasteur and Robert Koch at the end of the nineteenth century, the detection of viable bacteria has been carried out by cultivation and enumeration of colony forming units (CFU). Practically all judgments on hygiene, food safety, conservation treatments, drinking water quality, infections of pathogens, efficacy of disinfectants and antibiotics are based on growth on solid agar medium followed by CFU counts. However, the assessment of cell viability on agar plates is laborious, it requires at least an overnight incubation and the outcome yields little information on bacterial physiology. Besides, the assessment of CFU counts is limited to bacteria that are readily culturable under laboratory conditions and even when they are, the failure of bacteria to reproduce on an agar plate does not necessarily imply that they are metabolically inactive or were inactive at the time of sampling [1,2].

To overcome the shortcomings of CFU enumeration pointed out above, a number of alternative, cultivation-independent methods have been applied over the years to get hold of indicators for bacterial cell viability. The most commonly employed methods include fluorescence-based assays for enzymatic activity, electron transport and membrane permeability and molecular approaches for the detection of rRNA or specific mRNA molecules. The fluorescence-based assays provide signals that can be related to known cell properties, which can be assessed at the cellular rather than population level by the use of fluorescence microscopy and flow cytometry. The molecular approaches include microarrays and real-time PCR to select and quantify specific RNA molecules. The latter methods have been generally looked upon as providing indicators of specific aspects of bacterial physiology rather than indirect measures for bacterial cell viability valid under a wide range of lethal stress conditions. However, the feasibility of cultivation-independent biomarkers under a number of well-defined conditions as indirect indicators for cell viability is still a matter of debate [1,3,4].

In this study, we evaluated a number of commonly applied cultivation-independent methods under accurately defined conditions by a gradual exposure of the Gram-positive model bacterium *Bacillus subtilis *to heat stress. We systematically determined the effects of heat exposure on two cultivation-dependent measures, the ability to form colonies on agar plates, expressed in CFU counts, and outgrowth of the heat-exposed bacteria diluted in liquid cultures, expressed in the time delay to reach mid log phase. Furthermore, we determined a number of cultivation-independent measures, including the permeability of the bacterial membrane for fluorescent compounds, the stability of ribosomal RNA molecules and the presence of specific gene transcripts, as identified with microarrays and quantitative PCR methods. The results presented here reveal clear correlations between viability and cultivation-independent indicators in *B. subtilis *exposed to lethal heat stress. Notably, the genome wide expression profiles of heat-exposed *B. subtilis *reveal a coherent transcriptional program including known heat shock responses [5,6] and the rapid expression of sporulation and competence genes, the latter only known to be expressed in the stationary growth phase [7,8]. This and other forms of coordinated gene expression in the bacterial cell continued even after cell death, in other words after all bacteria permanently lost their ability to reproduce.

## Methods

### Strains and culture conditions

The *B. subtilis *wild-type strains used in this study include the laboratory strains 168 1A1 (Bacillus Genetic Stock Center) and *B. subtilis *food product isolates A163 [9] and MC85 (see table 1) [10]. Strains were cultured by inoculation of a Tryptic Soy Agar (TSA) plate from a glycerol stock stored at – 80°C in 40 ml Tryptic Soy Broth (TSB) under agitation (100 RPM) at 37°C. TSA and TSB were supplemented with selective antibiotics, if applicable: 5 μg ml^-1 ^chloramphenicol (Cm); 6 μg ml^-1 ^tetraclyclin (Tc); 100 μg ml^-1 ^spectinomycin (Sp) and 1 μg ml^-1 ^erythromycin (Em) all derived from Sigma Aldrich Inc.

**Table 1:** *B. subtilis *strains used in this study

***B. subtilis *strains**	**Characteristics**	**Donator/reference**
168 1A1	*trpC2*/parental strain in this study	Bacillus Genetic Stock Center
A163	food product isolate	Stanley Brul/[9]
MC85	food product isolate	Stanley Brul/[10]
DS323	*sigD::tet*	Daniel Kearns [31]
PB344	*sigB::spc*	Chester Price/[32]
AH46	*spoIIAC::erm *(*sigF*)	Adriano Henriques
AH74	*spoIIGB::erm *(*sigE*)	Adriano Henriques/[33]
AH70	*spoIVCB::erm *(*sigK*)	Adriano Henriques
TNO2007.160	DS323 → 168 1A1 (*sigD*)	This study
TNO2007.164	PB344 → 168 1A1 (*sigB*)	This study
TNO2007.166	AH46 → 168 1A1 (*sigF*)	This study
TNO2007.167	AH74 → 168 1A1 (*sigE*)	This study
TNO2007.169	AH70 → 168 1A1 (*sigK*)	This study

### Heat treatment

An overnight culture of *B. subtilis *was diluted 20× in TSB to a volume of 600 ml and incubated at 30°C at 100 rpm until OD_600 _reached 0.5. Subsequently, portions of 40 ml of the preculture of *B. subtilis *were exposed to seven different temperature regimes in 250-ml bottles in a reciprocal-shaking water bath at 65°C. Duplicate samples were taken when the temperature in the bottles reached 40, 50, 57, 58, 59, 60, and 62°C (at incubation times of approximately 100, 200, 300, 320, 380, 500 and 720 s after transfer, respectively). Immediate quenching of metabolic activity in the samples was performed with cold methanol, according to described methods [11]. Briefly, 40-ml culture samples were sprayed in 160-ml of a stirred solution of 60% methanol, 66.7 mM HEPES, pH 6.5 that was kept at – 45°C in an ethanol bath. Besides RNA-isolations, the heat-treated cultures were directly subjected to outgrowth analysis to monitor the recovery time and flow cytometric analysis, as further described below. The outgrowth was carried out in 10× dilution series by OD measurements in time at 37°C in TSB by use of the Microbiology Reader Bioscreen C (Oy Growth Curves Ab Ltd, Finland). The time delay in reaching the mid log phase (OD wideband = 0.5) compared to the 30°C-control sample was used as a measure for the increased recovery time from heat injury for each specific exposure.

### RNA isolation

The RNA isolation protocol was adapted from an extraction method described previously [12]. In brief, cells were disrupted in a 2-ml screw-cap tube in the presence of 100-μm zirconium beads and SDS/phenol for 60 seconds in the Minibeadbeater 8™ (Biospec Products). After chloroform extraction, nucleic acids were precipitated, vacuum-dried, dissolved in distilled water and incubated for 15 min in the presence of DNase I (Qiagen). Finally, RNA was purified by phenol/chloroform extraction and ethanol precipitation as verified by absorption properties in the 200–300 nm region by the use of the ND-1000 spectrophotometer (NanoDrop Technologies, Wilmington, Delaware, USA). RNA MW profiles were verified with the 2100 Electrophoresis Bioanalyzer (Agilent Technologies). The RNA 6000 ladder (Ambion Inc, Austin TX, USA) consisting of 6 transcripts at a concentration of 20 ng/μl, ranging from 0.2 to 6.0 kb in length was used as a marker. In addition, a fast migrating "reference marker" compound was added to all samples for software alignment of all electropherograms within one LabChip run (Agilent Technologies).

### Construction of *B. subtilis *microarrays

The *B. subtilis *microarrays were constructed by the Micro-Array Department (MAD), University of Amsterdam. An oligonucleotide library of 65-mers (Sigma-Genosys Ltd.), which covered 4100 open reading frames of the *B. subtilis *168 genome [13], was spotted in duplicate on glass slides with a Generation III microarray spotter (Molecular Dynamics, Sunnyvale, California, USA) using Lucidea Spotting Pins (GE Healthcare) as described previously [14].

### Labelling of cDNA

Fluorescently labelled cDNA was prepared from 12.5 μg of total RNA by random hexamer pd(N)6-primer (Roche, Mannheim, Germany) polymerization using Superscript II reverse transcriptase (Life Technologies). Concentrations of nucleotides in labelling reaction mixture were 0.4 mM dATP, dGTP, dCTP and 0.2 mM dTTP. The final concentration of Cy3-dUTP or Cy5-dUTP (GE Healthcare) was 0.1 mM. Unincorporated Cy-dye-labelled dUTP, dNTP's, primers and salts were removed by purification with AutoSeq G50 columns (GE Healthcare).

### Hybridization and image analysis

Microarray slides were incubated for 45 min at 42°C with prehybridization solution (1% bovine serum albumin, 5× SCC and 0.1% SDS, filtered), washed three times with milliQ water, and dried by the use of a nitrogen flow. The Cy3-labelled cDNA from untreated cells sampled at 30°C, was mixed with Cy5-labelled cDNA from heat-treated cells for all hybridizations. After 2 min of denaturation at 95°C, hybridizations of microarray slides were performed overnight at 42°C in 40 μl of EasyHyb buffer (BioCat, Heidelberg, Germany) with both labelled cDNAs and 2.5 mg ml^-1 ^yeast tRNA (final concentration). Microarray slides were washed at room temperature for 10 sec in 1× SSC/0.2% SDS at 37°C, 10 sec in 0.5× SSC at 37°C, and twice for 10 min in 0.2× SSC at room temperature. Slides were dried by the use of a nitrogen flow and scanned with a ScanArray 5000 laser scanner (Perkin Elmer Life Sciences). The TIFF-images were quantified with the software package ImaGene version 5.6.1 (BioDiscovery, El Segundo, CA, USA).

### Data processing and normalization

The spot intensities of *B. subtilis *oligonucleotide microarrays were processed by the use of Microsoft Office Excel 2003 SP2. The intensities of 8200 spots were calculated by extraction of the median background (B) from the median signal (S) and values for S-B < 1 were set to 1. The raw Cy5/Cy3 ratios (r) were calculated from the remaining background-corrected median signals. The total intensity normalization was carried out by the normalization factor N, defined as the average Cy5 (S-B) divided by the average Cy3 (S-B)). The normalization factor was determined from spot intensities with S/B > 2 for *both *Cy5 and Cy3 channels in order to avoid bias resulting from the degradation of the major of the transcripts at temperatures from 57–62°C. The normalised Cy5/Cy3 ratios (R = r/N) with S/B > 2 for either the Cy5 or Cy3 channel were calculated. Ratios from duplicate spots on the array and 2 independent experiments were averaged and ^2^log transformed. Next, all gene IDs with less than 2 observations out of 8 conditions (30, 40, 50, 57, 58, 59, 60, 62°C) were removed from the data set, leaving 3850 out of 4100 gene IDs (94%). Expression profiles were analyzed with K-means clustering into 4 groups with the Pearson correlation as distance metric by the use of Multiple Experiment Viewer MeV v4.1 [15]. Subsequently, the ^2^log ratios of spot intensities were selected which showed 5 or more useful ratios out of 8 temperature conditions, comprising a total of 3562 ratios (87%). In addition, all determined transcription levels for each gene at each temperature condition were analyzed by the use of the T-profiler , a tool that uses the *t*-test to score changes in the average activity of predefined groups of genes [16,17]. Group definitions were obtained from the DBTBS , the database of transcriptional regulation in *B. subtilis*, release 4.1 [18].

### Microarray data accession number

Microarray data were deposited in the Gene Expression Omnibus (GEO) database  under accession number GSE12372.

### Flow cytometry

Heat-treated *B. subtilis *cell suspensions (OD_600 _= 0.5) in the temperature range of 40–62°C were directly labelled with propidium iodide (Molecular Probes Inc, Eugene, OR, USA) at 2 μM final concentration and incubated for 2 min at room temperature. For calibration purposes a sample treated for 10 min at 96°C (100% PI-permeable) was mixed with an untreated sample in relative amounts of 0, 20, 40, 60, 80 and 100% PI-permeable cells. For flow-cytometric analysis the cell-suspensions were diluted 40-fold in filtered phosphate-buffered saline solution and directly measured on a Coulter Epics XL-MCL flow cytometer (Beckman Coulter, Mijdrecht, The Netherlands) operating an argon laser (488 nm). For each sample, at least 10,000 cells were analyzed. Fluorescent signal data were collected in the FL4 detector channel, and the photomultiplier voltage was set at 917 V for all measurements. Data were captured and analyzed using the EXPO32 analysis software (Beckman Coulter). Figures were prepared for publication using MicroCal Origin (version 7.5).

### Fluorescence Microscopy

Labelling was carried out with fluorophores Syto9 and propidium iodide (PI) according to methods described in the LIVE/DEAD BacLight kit (Molecular Probes Inc.) [19]. Subsequently, 10-μl aliquots of the cultures were embedded in 1% (w/v) final concentration of agarose and deposited on the surface of a microscope slide. Samples were analyzed by the use of the BX61 TRF fluorescence microscope (Olympus Inc.).

### Quantitative PCR

The extraction of RNA was carried out on a cell suspension of 750 μl of bacterial cells, which were quenched immediately after heat exposures as described above in detail. For cDNA synthesis, 1 μg of the isolated total RNA was vacuum dried and incubated in 5 μl Random primer mix, consisting of 3 μl 5U μl^-1 ^RNAsin (Promega) and 2 μl 2 μg μl^-1 ^pd(N)6 random primer (Roche) for 5 min at 70°C. After cooling down for 10 min, 8.5 μl RT mix was supplemented: 1 μl RNAsin (10 U μl^-1^, Promega); 2.5 μl First Strand Buffer 5× (Invitrogen); 1 μl 0,1 M DTT (Invitrogen); 3 μl 2 mM dNTP's (Invitrogen) and 1 μl Superscript II enzyme (200 U μl^-1^, Invitrogen). The RT reaction was carried out for 60 min at 42°C. Subsequently, to degrade the RNA, 2.5 μl of 2.5 M NaOH was added to the reaction mix and incubated for 15 min at 65°C. This was followed by addition of 2.5 μl 2.5 M NaAc and 10 μl milliQ. The cDNA was purified by the use of a G50 Autoseq column, dried in vacuum and resuspended in 25 μl prior to the Q-PCR reactions.

Primer-probe combinations for Q-PCR were designed for the 23S rRNA, *dnaK*, *gspA *and *cotJA *gene transcripts by the use of Primer Express Software v2.0 (Applied Biosystems), as listed in Table 2. The Taqman probes contain the minor groove binder (MGB) probe in combination with a non-fluorescent quencher (NFQ) and a reporter (R). The experiment was performed using the 7500 Fast Real-Time PCR System (Applied Biosystems) with the following settings: 1 step of 2 min at 50°C and 10 min at 95°C, followed by 50 cycles of 15 seconds at 95°C and 1 min at 60°C. Dilutions of *B. subtilis *genomic DNA were used as quantitative standard (1 × 10^8 ^fg μl^-1 ^to 1 fg μl^-1^). Measurable RNA sample dilutions in the range of the standard were analysed and included in the assay to determine the level of genomic DNA contamination in the RNA preps. The composition of the Q-PCR mix included 15 μl 2× PCR Mastermix (Diagenode Liège Belgium) 1.3 μl Primer Forward (10 pmol μl^-1^), 1.3 μl Primer Reversed (10 pmol μl^-1^), 1.3 μl MGB Probe (5 pmol μl^-1^), 10 μl milliQ and 1 μl RNA sample.

**Table 2:** List of primer probe combinations for Q-PCR

**Name**	**Sequence**	**Primer Type**	**Amplicon size**
B.sub-gspA	ACAAAAGCCGCGTATTA	TAQMAN MGB	62 bp
B.sub-gspA-F	GCGGTTGAGAGCAGCCATA	Forward	
B.sub gspA-R	ATTAAGTCGGGAATCGAAATGC	Reverse	
B.sub-dnaK	AAGTTCGTTCAACTGCCGG	TAQMAN MGB	61 bp
B.sub-dnaK-F	TGAGCTTGGCGACGGTGTA	Forward	
B.sub-dnaK-R	CCCACCCAGACGGTTGTC	Reverse	
B.sub-cotJA	CGATGGAGGCTTTGAG	TAQMAN MGB	59 bp
B.sub-cotJA-F	GAGCACATGGAGCAGTTTTCG	Forward	
B.sub-cotJA-R	TCCTTCCAAAGGGTGCCTTT	Reverse	
B.sub-cheW	TACCGTACACGAAAGTGAA	TAQMAN MGB	63 bp
B.sub-cheW-F	TGGATGAAGCGAATGATGTGA	Forward	
B.sub-cheW-R	CGCCTTCTGGAGCAGATTCT	Reverse	
B.sub-23S	CGGTACAGAGTGTCCTAC	TAQMAN MGB	97 bp
B.sub-23S-F	GGCGAGCGAAACGGGAT	Forward	
B.sub 23-S-R	ACCTCTTCATCTACCTCGTTCCTTT	Reverse	

### Construction of *sigB*, *sigD*, *sigF*, *sigE *and *sigK *mutants

The DNA recipient strain *B. subtilis *strain 1A1 was cultured overnight in Spizizen salts minimal medium, containing per liter 14 g K_2_HPO_4_, 6 g KH_2_PO_4_, 2 g (NH_4_)_2_SO_4_, 1 g Na_3_C_6_H_5_O_7 _(sodium citrate) and 0.4 μg MgSO_4_, 0.2 g casamino acids, 5 g glucose (Tritium Microbiology) and 80 μg L-tryptophan (Sigma Aldrich Inc.). The overnight cultures were diluted 10 times in Spizizen salts minimal medium, and grown for 3 more hours under the same conditions. Subsequently, one volume unit of starvation medium (same as Spizizen salts minimal medium, but without casamino acids and L-tryptophan) was supplemented and growth was continued for two more hours. Competent cells were incubated for 15 min at 37°C in the presence of isolated genomic DNA from sigma factor mutants as listed in Table 1 and plated on selective agar media. The transformed *sigF*, *sigE *and *sigK *strains were checked for the loss of ability to sporulate after overnight growth in nutrient agar (Tritium Microbiology) supplemented with 0.5% (w/v) CaCl_2_.H_2_O and 0.5% (w/v) MnSO_4_. The *sigD *strain was checked for loss of motility by microscopy.

## Results

### Heat exposure regime

**Figure 1:**
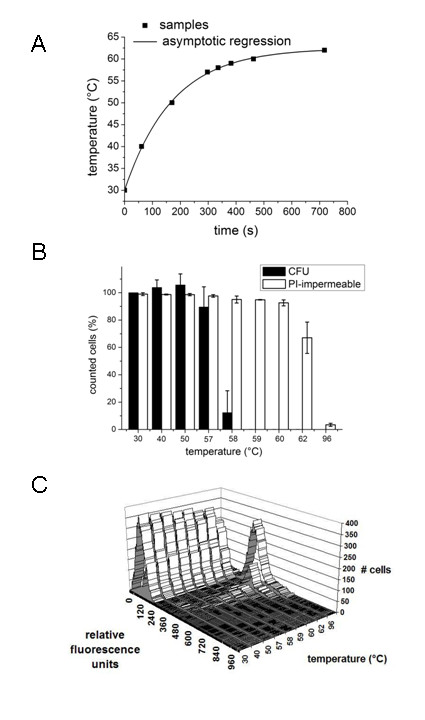
**Effect of lethal temperature treatment**. A) Temperature regime applied to exponentially growing *B. subtilis *cells. Duplicate samples were taken when the temperature reached 40, 50, 57, 58, 59, 60, and 62°C (indicated by black squares). Quenching of metabolic activity in the samples was performed by addition of cold methanol. B) Membrane integrity of *B. subtilis*. Heat-treated *B. subtilis *cell suspensions were directly labelled with propidium iodide, followed by flow-cytometric analysis. The fluorescence intensity per event of at least 10,000 cells per sample is shown as a histogram for each temperature treatment. C) Culturability versus membrane integrity. Colony forming units (CFU) of *B. subtilis *were determined after increasing periods of heat treatment (filled bars). In addition, the percentage of non-fluorescent cells after propidium iodide staining is indicated (open bars).

Here we present a systematic comparison between cultivation-based assessment of cell viability and cultivation-independent indicators for cell viability. As such comparisons require strictly defined conditions [1], we carefully selected conditions allowing for a precise and reproducible monitoring of the transition between viable and nonviable bacteria. Throughout this study we consider the definition for viability identical to that for culturability, as justified for readily culturable bacteria such as *B. subtilis *(see [1] for an extensive discussion on definitions for bacterial cell viability). Lethal heat stress was applied to *B. subtilis *in a tightly controlled manner in order to obtain a well-defined and gradual transition from viable to nonviable cells. First, the temperature sensitivity was investigated for *B. subtilis *cells exposed to a wide range of temperature treatments. Batch cultures of *B. subtilis *cells incubated at a number of constant temperatures indicate that growth still occurs up to 54°C, while no growth was observed at temperatures higher or equal to 56°C (data not shown). Next, we selected a lethal incubation temperature of 65°C based on CFU counts, which allows monitoring the transition from a living to a heat-inactivated bacterial cell culture within 15 minutes. The experimental set-up included the transfer of a shake flask containing a bacterial cell culture from 30 to a 65°C water bath. The temperature in the shake flask was continuously monitored as a function of time and samples were taken at predefined temperatures in the range from 30 to 62°C over a total time span of 12 minutes for all cultivation-dependent and independent analyses described throughout this study. The temperature profile as well as the exact time at which the samples have been collected for further analyses has been indicated in Figure 1A.

### Colony forming units

The number of colony forming units was determined for heat-exposed bacterial cell suspensions by plating dilution series on TSB agar plates, followed by enumeration of colonies after overnight incubation. At temperatures above 57°C significant loss of cell viability was observed, including a reduction of almost 1 log unit at 58°C (Figure 1B) and more dramatic reductions of 3, 4 and 6 log units at 59, 60 and 62°C, respectively (Table 3). The heat injury of bacterial cells may lead to relatively long recovery times before colonies appear. Thus, we carried out CFU counts after 24, 72 and 84 hours of incubation at 37°C. The counts at 72 and 84 hours did indicate in some cases increased levels, but did not exceed more than 10% of the initial CFU count at 24 hours (data not shown). Therefore, we further neglected the slight underestimation of CFU counts at 24 hours and used the 24 hour-counts throughout the study. We checked whether the death rate of the 1A1 laboratory strain was representative for the *B. subtilis *species by testing the *B. subtilis *natural isolates A163 and MC85 (Table 1). We confirmed that the survival curves were very similar for all *B. subtilis *strains tested (data not shown). Furthermore, we checked for the presence of *B. subtilis *1A1 spores in TSB growth medium throughout this study by microscopic inspection as well as CFU counts after heat-inactivation of vegetative cells, showing spore fractions ranging from 0 to 0.0001 (data not shown), too low to interfere with the studies described here.

**Table 3:** Culture-dependent and independent viability indicators

					**^2^log ratios μ-array**			**^2^log ratios q-PCR**			
**T(°C)**	**CFU(^10^log)**	**LT(h)**	**PM (%)**	**16S/23S**	***dnaK***	***gspA***	***cotJA***	***dnaK***	***gspA***	***cotJA***	***23S rRNA***
30	8.3	0	0	0.9	0.1	-	-0.2	0.0	0.0	0.0	0.0
40	8.4	0.2	1	0.9	0.5	0.9	0.0	0.2	3.9	-0.3	-0.3
50	8.4	0.8	1	0.7	2.0	6.1	0.4	1.1	5.5	1.2	0.0
57	8.2	4	2	0.4	2.4	4.3	1.2	0.0	4.8	1.1	-0.1
58	7.7	8	3	0.3	1.6	1.8	2.9	-1.0	3.7	1.8	0.0
59	5.1	9	5	0.2	0.6	0.8	3.8	-1.9	3.8	2.7	0.2
60	4.3	9	6	0.2	-1.0	-0.4	4.5	-2.9	0.0	2.5	0.4
62	2.5	12	25	0.1	-2.0	-1.2	4.8	-4.1	0.6	3.3	0.4

Listed in columns is the applied temperature, the number of colony forming units (CFU) in ^10^log units, the lag time (LT) of the growth curves in hours (h), the number of cells with a propidium iodide permeable membrane (PM) expressed in percentage (%), the ratio between 16S and 23S ribosomal rRNA as determined by gel image analysis, the ^2^log ratios of expression levels at the indicated temperature in the first column and the reference temperature at 30°C for *dnaK*, *gspA *and *cotJA *genes (determined by microarrays), and the ^2^log ratios of expression levels at the indicated temperature in the first column and the reference temperature at 30°C of transcripts for *dnaK*, *gspA*, *cotJA *and *23S rRNA *genes (determined by quantitative PCR with primer probe combinations listed in Table 2).

### Outgrowth in liquid cultures

The recovery of bacteria from each temperature exposure was determined in dilute liquid cultures in triplicate by monitoring the time delay in reaching the mid log phase (OD wideband = 0.5) compared to the 30°C-control sample. Small increases in the average lag time were observed (0.2 and 0.8 hours) for temperature exposures of 40 and 50°C (Table 3). However, their significance remains uncertain as standard deviations for observed lag times ranged from 0.2 to 1.5 hours. A more drastic and significant change in the lag time of approximately 4 hours occurred at a temperature of 57°C, while at this temperature no significant loss of CFU was observed (Figure 1B). Apparently, the cells are able to fully recover from temperature treatments up to 57°C during an extended incubation in growth medium at 37°C. For more stringent temperature treatments in the range of 58 to 62°C the lag time ranged from 8 to 12 hours (Table 3). The relatively large increase in lag time from 4 to 8 hours for temperature treatments of 57 and 58°C coincides with the first loss of CFU at the latter temperature. Thus, for temperatures from 40 to 57°C the increase in lag time exclusively results from cellular recovery processes, while the increase in lag time at 58°C and higher results from both cellular recovery processes as well as the loss of viable cells in the culture. These results clearly show that the time delay to reach the mid log phase is a more sensitive measure to assess the effect of a heat exposure than CFU counts.

### Membrane integrity

The effect of heat on *B. subtilis *cells was also studied with a number of cultivation-independent methods, including the fluorescence-based BacLight kit (Molecular Probes Inc.). This kit employs two nucleic acid stains, the green-fluorescent SYTO9 and the red-fluorescent propidium iodide stain, which differ in their ability to permeate bacterial cells. The cellular membrane is in general permeable to SYTO9, while propidium iodide penetrates only bacteria with damaged membranes. Propidium iodide reduces SYTO9 fluorescence when both dyes are present together. Thus, bacteria with intact membranes fluoresce green, while bacteria with damaged membranes fluoresce red [19]. Microscopic inspection confirmed that this system can be applied to stain heat-exposed *B. subtilis *cells (data not shown). However, we found that the fluorescence of the SYTO9 fluorescent dye was relatively unstable under the conditions used, as the green fluorescence signal slowly decayed, while the fluorescence of the propidium iodide dye remained stable. Therefore, we subjected bacteria that were stained only with propidium iodide to flow-cytometric analysis. A calibration curve of mixtures of living and heat-killed bacteria (10 min at 96°C) showed a perfectly linear correlation between the percentage of heat-exposed bacterial cells (0, 20, 40, 60, 80 and 100%) and the number of events with relatively high red fluorescence intensity (data not shown). The heat-exposed bacterial cell samples were stained with propidium iodide and analyzed by flow-cytometry. The three-dimensional representation in Figure 1C shows the number of cells plotted as a function of fluorescence intensity in the red FL4 channel for each temperature treatment. Clearly, the heat-induced decrease of non-fluorescent cells coincides with an increase of membrane-permeable red-fluorescent cells all with Gaussian-shaped population distributions. The effect is most prominent at 62°C (apart from the control experiment at 96°C), at which 25% of the cell population is permeable to the propidium iodide dye. A comparison of CFU counts with data from propidium iodide stained bacteria (Figure 1B, C and Table 3) shows that the membrane integrity is a much more insensitive measure for the applied heat regime than the ability to form colonies. In the range of 59–62°C the majority of bacteria do still contain an intact membrane, while more than 99.9% of these bacteria lost their ability to form colonies. Apparently, heat-induced cell death precedes the loss of membrane integrity.

### Ribosomal RNA stability

**Figure 2:**
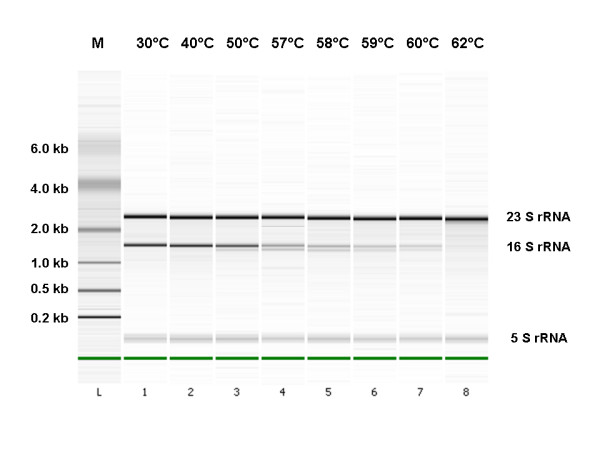
**Electrophoretic analysis of total RNA extracts**. Purified RNA samples of *B. subtilis *cells, heat treated with a temperature profile as plotted in Figure 1A, were examined by the use of the 2100 electrophoresis BioAnalyzer (Agilent Technologies). Major ribosomal RNA bands are indicated (23S, 16S and 5S rRNA). M = molecular weight DNA marker. The green bands indicate the fast migrating "reference marker" compound for software alignment of all electropherograms within one LabChip run.

In order to study the effect of extreme heat stress at a molecular level, we evaluated the heat stability of 16S and 23S rRNA molecules. The heat stability of total RNA isolations from bacterial cells was assessed in a number of independent experiments by the use of the 2100 Electrophoresis Bioanalyzer (Agilent Technologies). The 16S/23S ratios indicated in Table 3 were calculated from the integrated peak intensities. The temperature treatments in the range from 30–62°C did not result in systematic changes in the total yield of RNA as determined by 260 nm OD measurements (data not shown), but the electrophoresis patterns clearly indicated that heat-induced degradation of 16S rRNA starts at exposures from 50°C. However, the 23S rRNA appears resistant against the heat treatment over the entire temperature range up to 62°C, evident from the discrete bands of the same intensity over the entire temperature range (Figure 2). The levels of 16S rRNA decreased to less than 10% of the 23S rRNA level (Table 3). The appearance of faint but discrete bands in the range from 50–62°C at molecular weight positions lower than that of the 16S rRNA transcript suggests that heat-induced degradation of this RNA molecule occurs at least partly through breakage at preferential sites. In addition, the 5S rRNA molecule seems quite heat stable, since a discrete band remains present throughout the temperature treatment. Prolonged heat treatments at temperatures higher than 62°C led to complete degradation of ribosomal RNA, including 23S rRNA, and eventually also DNA (data now shown).

### Genome-wide expression profiles

**Figure 3:**
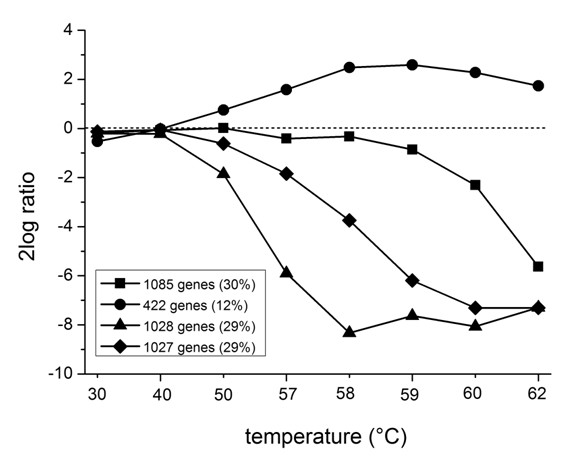
**K-means clustering of genome-wide expressiondata**. Expression profiles were analyzed by the use of K-means clustering-classifying the data set into 4 clusters – showing the global behaviour of gene transcripts during the heat exposure from 30 to 62°C. The 8 data sets include the temperature exposures (from left to right) of 30, 40, 50, 57, 58, 59, 60, and 62°C.

The genome-wide transcriptional responses of *B. subtilis *to lethal heat stress were analyzed by microarrays that represent 4100 open reading frames covering the entire genome of this organism [13]. Labelled cDNA probes of the temperature of interest (Cy5-labelled) and the 30°C reference temperature (Cy3-labelled) were mixed for every hybridization experiment. Independent heat-exposure experiments were carried out to obtain replicates. Besides, experiments were carried out with RNA isolated from two *B. subtilis *food product isolates (A163 and MC85) (Table 1). All data were expressed as ^2^log ratios of treatment and reference. The data processing filters (for details see Materials and Methods) did not result in a systematic loss of data points (number of genes with useful ratios) upon increasing temperatures. First, a k-means clustering analysis – a procedure to classify a given data set through a certain number of clusters (assume k clusters) fixed a priori [20] – was applied to study the global behaviour of gene transcripts during the heat exposure from 30 to 62°C (Figure 3). We found that clustering with a minimum of 4 groups of genes leads to a representative description of global gene expression behaviour. Three of the gene clusters each cover approximately 30% the expression data and show decreased transcript levels at elevated temperatures decaying at similar decay rates, but starting to decrease from 50, 57 and 60°C, respectively (Figure 3). This major part of transcript ratios (88%) decreases to values below ^2^log values of -5 at elevated temperatures as a result of the heat treatment. The remaining 12% of the transcripts representing the fourth cluster is induced from 50°C and still present at reference or elevated levels, even under conditions where cell viability has been completely lost.

### Heat-induced expression of gene clusters

**Figure 4:**
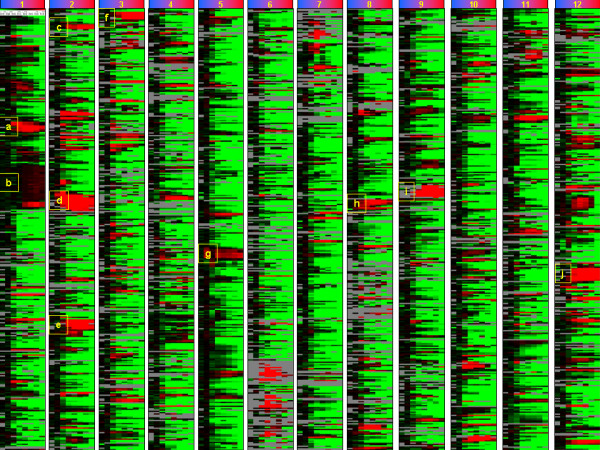
**Genome-wide expression profiles**. Presentation of the ^2^log ratios of expression profiles of all 4100 open reading frames from 30 to 62°C. Color coding ranges from green (^2^log ratio = -3) via black (^2^log ratio is 0) to red (2log ratio is 3); the grey color indicates no signal with the filters applied. All 4100 open reading frames are sorted in the order present on the *B. subtilis *genome (from the first ORF *dnaA *to last *rprnH*) and represented over a total of 12 columns. Each column contains 8 sub-columns representing a temperature exposure range from the reference temperature of 30°C to the final temperature of 62°C, as indicated by the upper bars from blue to red.

**Figure 5:**
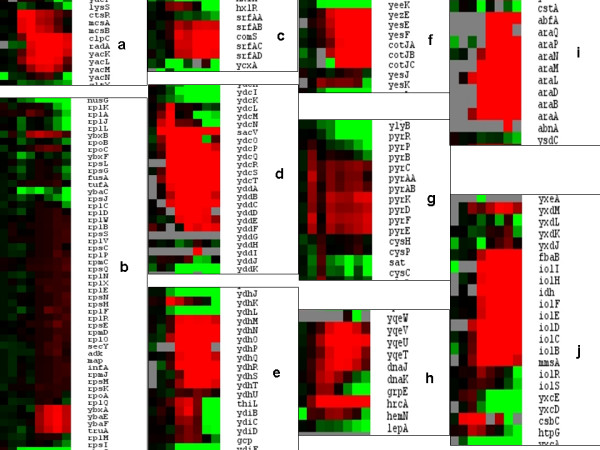
**Heat-induced expression profiles in gene clusters**. Presentation of ^2^log ratios of the gene clusters a – j. Color coding ranges from green (^2^log ratio = -3) via black (^2^log ratio is 0) to red (2log ratio is 3); the grey color indicates no signal with the filters applied. (a) the class III heat shock operon *ctsR*, *mcsA*, *mcsB*, *clpC*, the DNA repair gene *radA*, and genes of unknown function *yacK-N*, (b) *rplK-rpsI*, a region of 67 ORFs, encoding at least 47 ribosomal proteins, (c) the surfactin syntetase/competence genes *srfAB-srfAD*, (d) the mobile genetic element, ICEBs1, an integrative and conjugative element, (excision requires the integrase, *ydcL*, predicted to encode a tyrosine recombinase similar to that of phage lambda, and the excisionase, *sacV*), (e) the genes *ydhM-T *involved in carbohydrate metabolism, (f) the inner spore coat genes *cotJA-C *and upstream genes *yezE-yesF*, (g) pyrimidin biosynthesis gene cluster *pyrB-E*, (h) the class I heat shock regulon *hrcA-yqeV*, (i) the L-arabinose operon *araA-Q *and (j) the genes *iolB*-*I *involved in myo-inositol catabolism.

In order to obtain an impression of the cellular functions associated with genes in the fourth cluster, we applied two different supervised strategies. In the first, described in this paragraph, we selected for large, temperature-responsive, unregulated clusters of genes, while in the second strategy, described in the next paragraph, we evaluated the average expression level of genes known to be under control of a common transcriptional regulator. The identification of interconnected gene clusters, behaving in a similar manner in terms of expression profiles, can be very informative in bacteria, as functionally related genes are often in close proximity of each other and co-transcribed in polycistronic transcripts. All 4100 open reading frames were sorted in the order present on the *B. subtilis *genome (from the first ORF *dnaA *at 0.4 kb from the origin of replication to the last ORF *rpmH *at 4214.4 kb) and their ^2^log expression ratios were represented over a total of 12 columns (Figure 4). Each of 8 subcolumns within a column represents a different temperature exposure, from the reference temperature of 30°C to the final temperature of 62°C, indicated by the upper horizontal bars from blue to red. The 10 largest interconnected gene clusters (letters a – j), containing the highest average heat-induced gene expression ratios, were identified in Excel by iterative smoothing of ^2^log ratios over the genome (window size: 8) of the average of the 6 gene expression levels from 50 to 62°C. In Figure 5 these 10 clusters are isolated from their genome context and presented in more detail. The gene clusters include (a) the class III heat shock operon (b) a stretch of 67 ORFs containing at least 47 genes encoding ribosomal proteins, (c) the surfactin biosynthesis operon/competence genes, (d) the mobile genetic element ICEbsl, (e) an operon involved in sugar/carbohydrate metabolism, (f) an operon encoding inner spore coat proteins, (g) the pyrimidine biosynthesis operon, (h) the class I heat shock operon, (i) the L-arabinose operon and (j) the operon involved in myo-inositol catabolism. We further substantiated these results by verifying the heat-induction of these gene clusters in the natural *B. subtilis *strains A163 and MC85 (Table 1). We confirmed the induction of all 10 clusters in these isolates, except for the mobile genetic element ICEbsl, which is most probably absent in both strains as no expression signals were observed at all for the genes present in this clusters. Interestingly, only a minority of the above gene clusters has been identified as part of the classic heat-shock response to less stringent, sub-lethal heat treatments [5,6].

### Heat effects on transcriptional regulation

**Figure 6:**
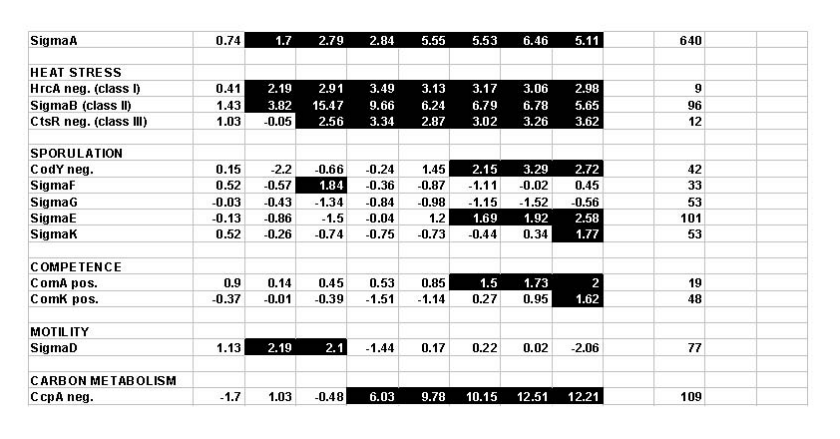
**T-profile analysis of transcription regulons**. Transcription profiles for each temperature condition were analyzed by the use of the T-profiler, a tool that uses the *t*-test to score changes in the average activity of predefined groups of genes [16]. Group definitions were obtained from the DBTBS: the database of transcriptional regulation in *B. subtilis*, release 4.1 [18]. A total number of 9 categories were displayed as function of temperature (the 8 samples from 30–62°C as in Figure 1A). T-values ≥ 1.5 are indicated in black (arbitrarily selected T-value). The right column indicates the total number of genes of the regulon included in the analysis.

A second approach to improve understanding in gene expression at lethal heat stress temperatures is through the use of previously identified transcriptional regulators. This type of analysis was carried out with T-profiler, a tool that uses the *t*-test to score changes in the average activity of genes under control of transcription factors, including σ-factors and other known activators and repressors [16]. In this study we used the DBTBS database [18], containing a collection of 116 experimentally validated transcription factors and 1253 gene regulatory relations, to evaluate the role of these regulators during the applied heat regime. The first and largest pre-defined group of genes (average over a total of 660 genes) up regulated at elevated temperatures consists of the genes under control of the house-keeping sigma factor σ^A ^(Figure 6). This is in agreement with its control by a heat sensitive promoter [6]. In addition, three classes of heat shock genes have been identified. We found the induction of the heat shock stimulon, consisting of the major class I, II and III heat shock regulons, under control of the regulators HrcA, σ^B ^and CtsR, respectively. The σ^B ^or general stress response transiently peaked at 50°C, while the overall expression of the HrcA and CtsR regulons remained fairly constant after induction over the entire heat regime (Figure 6).

Besides the classical heat shock responses, we identified a number of novel responses to heat stress. Notably, the response induced at the lowest temperature, already maximal at 40°C, includes the expression of genes under control of σ^D^, the transcription factor for motility. This response is only present at 40–50°C; it involves among others the *fla/che *operon, the *lytA-C *genes for autolysis and genes encoding methyl accepting chemotaxis proteins *mcpA*-*B*. Surprisingly, the heat-induced transcriptional response also includes a number of sporulation and competence genes, described in more detail below. One of the most prominent responses at high temperature was the derepression of large regulons involved in carbon metabolism, controlled by the CcpA (average of 109 genes), AraR (regulator of the arabinose operon; average of 12 genes) and IolR (regulator of the inositol metabolism; average of 13 genes) transcription factors. Note that both the inositol and arabinose operons are controlled by CcpA, a global regulator that directs the carbon flow (Figure 6).

**Figure 7:**
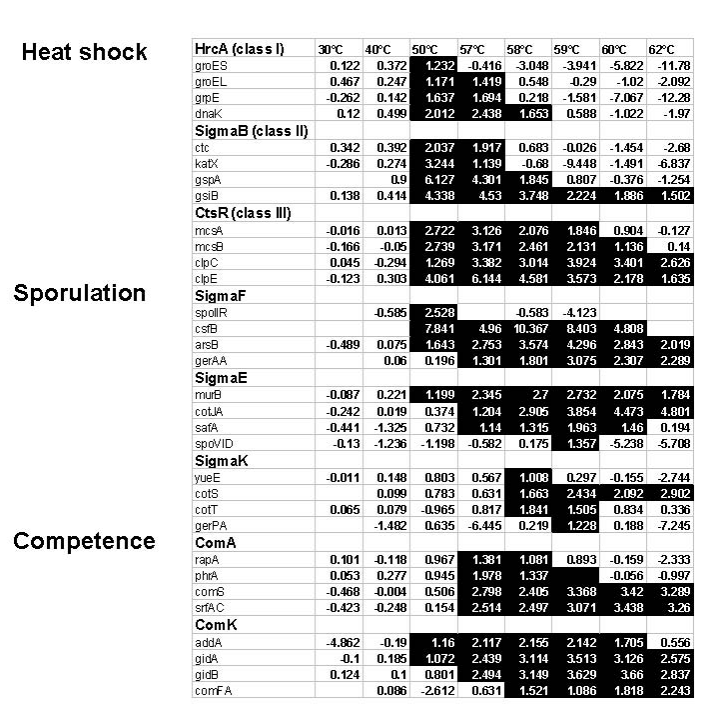
**Heat-induced expression levels of selected genes**. The ^2^log ratios of representative, up-regulated genes under control of heat shock, sporulation and competence transcription regulators. ^2^Log ratios ≥ 1 are indicated in black (arbitrarily selected value).

In order to clarify the T-values obtained for an interesting sub-set of the regulons described above, we depicted the ^2^log ratios for a number of representative genes, which are part of the regulons for the heat shock responses, sporulation and competence development in Figure 7. For reference, the ^2^log ratios have been indicated showing the heat-induction of 4 representative genes of the HrcA, σ^B ^and CtsR heat shock regulons, also described in a large number of previous studies [5,6,21,22]. Next, the ratios for 4 representative genes of regulons controlled by 3 sporulation transcription factors have been depicted. The transcriptional program for sporulation includes an asymmetric cell division and takes approximately 8 hours under laboratory conditions. During the process of sporulation, groups of genes are sequentially activated and silenced, representing distinct intermediate developmental stages [8]. There are 4 known sporulation specific sigma factors, σ^E^, σ^F^, σ^G ^and σ^K ^that cascade sequential and compartment-specific gene expression in mother cell [23] and forespore [24]. The σ^F ^factor becomes active only in the forespore and σ^E ^only in the mother cell, followed by σ^G ^and σ^K^, respectively. The timing of σ^F ^is controlled by the SpoIIAA/AB/E regulatory system. In this study, we observed the rapid heat-induced induction of genes under control of σ^F^, σ^E ^and σ^K ^on a time-scale of minutes rather than hours. The observed transcriptional response involved the up regulation of only a minor, but significant part of the sporulation genes, with a sequence of gene activation matching that of the normal sporulation program in the mother cell. Up regulated genes under regulation of sporulation transcription factors include the σ^F^-controlled genes *bofC*, *csfB*, *arsB*, *gerAA *and *spoIIR *and a number of genes under the dual control of σ^B ^and σ^F^. Interestingly, the *spoIIR *gene product is a signaling molecule that presumably activates the SpoIIGA protease leading to the processing of pro-σ^E ^into σ^E ^in the mother cell. The σ^E^-controlled genes include *murB*, *murG*, *divIVB*, *mmgD*, *mmgE*, *yesK *and the genes encoding the inner spore coat proteins *safA*, *cotJA*, *cotJB*, *cotJC *and *spoVID*. Finally, we noted activation of σ^K^-controlled genes *cotS*, *cotT*, *gerPA*, *yueE *and the genes under the dual control of σ^E ^and σ^K ^y*itD *and *spsG *(Figures 5 and 7).

We observed a comparable partial activation for genes involved in competence development. Competence is another stationary-phase differentiation process leading a cell-type to allow uptake of foreign DNA. The transcription factor for competence is ComK, which is post-translationally activated by the product of the *comS *gene, which is part of large operon that encodes surfactin biosynthesis and is in turn under control of the ComP/ComA two component system [7]. Again only a minor sub-set of competence related genes was involved, including *comS*, *srfAB*, *srfAC*, *srfAD*, *phrA *and *rapA *under control of ComA and *addA*, *addB*, *gidA*, *gidB*, *comFA*, *comGG*, *thdF*, *yyaA *controlled by ComK (Figures 5 and 7).

### Validation of transcript levels by quantitative PCR

Microarray results have been validated by the use of quantitative PCR. We selected the 23S rRNA gene as an internal standard based on its stability under the experimental conditions used in this study (Figure 2). In addition, we selected the *cotJA *gene, which encodes a spore coat protein, under control of transcription factor σ^E^, which is known to be active during sporulation in the mother cell. Furthermore, the *dnaK *gene, which encodes a class I heat shock gene, a chaperone, under control of the transcriptional repressor HrcA. Finally, the *gspA *gene, which encodes the general stress protein GspA and is a general reporter gene for activity of the general stress response transcription factor σ^B ^[25]. The Q-PCR analysis shows a good match between levels of heat-induction resulting from microarray and Q-PCR analysis of three typical heat-induction profiles (compare Figures 5, 7 and Table 3). The Q-PCR results of *gspA *transcripts confirm the transient induction of the σ^B^-controlled genes at 40 and 50°C, the more stable induction of *dnaK *transcripts at elevated temperatures and the gradually increasing levels of *cotJA *transcripts as a representative of the σ^E ^regulon.

### Assessment of *cotJA *transcript levels in σ-factor mutants

The heat-induced expression levels of the *cotJA *gene were verified in *B. subtilis *null mutants for the sporulation-transcription transcription factors σ^F^, σ^E^, and σ^K^. In addition, we checked expression effects of the same gene in null mutants of the heat-activated, general stress response sigma factor σ^B ^and the motility transcription factor σ^D ^(negative control). The results in table 4 clearly show that the heat-induced expression of *cotJA *is dependent on the presence of sporulation-specific sigma factors σ^F ^and σ^E^, but not on the sigma factors σ^B^, σ^D ^and σ^K^. These results are in line with the current model for the sporulation program, including the control of *cotJA *by σ^E^, which is in turn activated by an σ^F^-controlled gene, while σ^K ^plays a role in the signalling cascade further downstream. Null mutations of the heat-transcription factors σ^B ^and σ^D ^do not affect the heat-induction of *cotJA*. Notably, the expression levels of *cotJA *are much higher at the 30°C reference temperature in the absence of σ^F ^and σ^E^. This can be explained from the loss of expression of σ^F ^and σ^E ^controlled-genes that repress *cotJA *expression under non-sporulating conditions; mechanisms of repression and derepression have been found to play an essential role in the sporulation signal transduction cascade, but have not been specifically observed for control of the *cotJ *operon [23].

**Table 4:** Heat-induced expression levels of *cotJA *in *B. subtilis *σ-factor mutants

**strain**	**EL_30°C _(pg)**	**EL_60°C _(pg)**	**^2^log R**
168 1A1 (*wt*)	0.02	0.50	4.7
TNO2007.164 (*sigB*)	0.01	0.52	5.6
TNO2007.160 (*sigD*)	0.03	0.59	4.2
TNO2007.166 (*sigF*)	0.77	0.05	-3.8
TNO2007.167 (*sigE*)	0.48	0.06	-2.9
TNO2007.169 (*sigK*)	0.04	0.77	4.3

Listed in columns is the *B. subtilis *strain, the *cotJA *gene expression level at 30°C (EL_30°C_) expressed in picograms (pg) *B. subtilis *chromosome equivalents, normalized to the amount of isolated *23S rRNA *transcript, the *cotJA *gene expression level at 60°C (EL_60°C_) expressed in picograms (pg) *B. subtilis *chromosome equivalents, normalized to the amount isolated of *23S rRNA *transcript, and the ^2^log ratios of expression levels at 60°C (column 2) and the reference temperature at 30°C (columns 1). All values listed in this table were obtained by quantitative PCR with primer probe combinations listed in Table 2.

## Discussion

In this study we subjected the Gram-positive bacterium *B. subtilis *to a lethal temperature regime and analyzed the effects of the treatment in a comparative analysis using a number of cultivation-dependent and independent methods. The application of cultivation-independent methods as an alternative to assess bacterial viability is highly valuable for a number of reasons. A rapid measurement of the physiological state of the bacterium indicating whether it survived a certain processing condition (heat, high pressure, antimicrobial compounds) would save enormous amount of time and labour, currently lost in the classical routine of plating and counting. The timely and laborious plating method renders high-throughput screenings an almost impossible mission, although the recently reported miniaturization of the culture plate to a micro-Petri dish holds great promise for the future [26].

Any cultivation-based method results by definition in an estimate of the survivors *after *a treatment under predefined growth conditions, which are usually very different from those present during the actual processing conditions. A rapid measurement of the physiological state of the bacterium would be beneficial, as it reflects the direct cellular response towards processing conditions and could yield information on the successful survival strategy of the bacterium. This could allow a rational design for preferred adaptations in processing conditions aimed at a more efficient bacterial inactivation for harmful microbes or survival for beneficial ones.

The need for cultivation-independent methods to assess bacterial cell viability is most evident when microbes only grow in their natural habitat and colony formation is absent or extremely slow in any of the available growth media. Likewise, the enumeration of colonies is not applicable for bacteria present in dense populations of multiple species for which no selective media are available. In those cases the highly selective PCR-based assessment of specific gene transcripts, as further discussed below, can be applied to assess cell viability of one bacterial species in a complex population.

The workflow in this study starts from the assessment of bacterial viability upon exposure to a lethal heat treatment with conventional methods, including the enumeration of colonies, the determination of outgrowth curves and the widely used dead/live stain in the *Bac*Light kit [19]. These experiments set a clear reference for molecular studies discussed below and roughly establish the 40 to 57°C temperature regime as inhibitory and sub-lethal, as judged from the extension in the lag phase and CFU counts (Table 3). The membrane integrity as judged from the dead/live stain is clearly a less sensitive indicator for the subtle heat regime applied here than colony enumeration or lag phase extension (Table 3), but more stringent temperature treatments do lead to a 100% dead (propidium-iodide permeable) cell population (Figure 1C).

The electrophoretic analysis of total RNA extracts clearly indicates a much higher heat tolerance of 23S rRNA molecule for the applied heat regime than 16S rRNA molecule, as one can assume that constant level of 23S rRNA cannot exclusively result from higher 23S rRNA synthesis rates (Figure 2). The difference in heat susceptibility of 23S and 16S rRNA molecules is commonly observed among prokaryotes, including *B. subtilis, Staphylococcus aureus *and *Salmonella typhimurium *[27-29]. The results clearly show that *B. subtilis *is able to fully recover from the observed heat-induced rRNA degradation, as significant cell death only occurs from 58°C exposures, while a major part of 16S rRNA has been degraded at 57°C (Table 3). Therefore, the level of 16S rRNA is not a very good indicator for cell viability under these conditions.

A representation of the overall behaviour of mRNA transcripts during the heat treatment shows a great variability in expression levels of genes, with the majority of transcripts completely degraded at higher temperatures (Figure 3 and 4 and additional file 1), the time-lapse movie of heat-induced expression profiles). An overview of all the gene expression levels ordered according to the corresponding gene position on the circular *B. subtilis *genome allowed the selection of 10 major clusters of gene transcripts, which were highly up regulated and most of them stable over the entire heat regime from 50–62°C (Figure 5). The identified gene clusters matched many of those identified by the T-profile analysis, which uses pre-defined groups of genes known to be co-regulated (Figure 6). Strikingly, there is one very large cluster of genes, which show very similar transcription levels during the entire heat treatment (cluster b, Figure 5). These transcripts are encoded by a stretch of 67 ORFs containing at least 47 ribosomal protein genes. Apparently, the regulation and stability of the transcripts encoded by these house-keeping genes is such that they remain more or less constant during the applied heat regime, in accordance with their essential function in protein synthesis.

In addition, gene clusters were identified containing the classic heat shock operons class I and class III (h, a, Figure 5), while the class II heat shock or σ^B ^regulon was only identified in the T-profile analysis as a result of the widespread distribution of genes from this regulon over the genome. The cluster of genes known to be highly induced upon stress is the mobile genetic element ICEbs1, which up regulation occurs upon global DNA damage (cluster d) [30]. This fact and the up regulation of the cluster of pyrimidine genes (cluster g) may be part of the cell's response towards heat-induced DNA damage. The gene clusters (e, i, j) which are all involved in catabolic carbon metabolism may indicate the cell's attempt to deal with energy stress and generate ATP. Most surprising is the up regulation of a number of genes, known to be involved in stationary phase processes sporulation (cluster f) and competence development (cluster c). As their activation is usually strictly controlled by dedicated transcription regulators, we tested whether the observed up regulation of inner spore coat genes (*cotJ *operon) is dependent on sporulation-specific transcription factors. We confirmed that the heat-induced expression of the *cotJ *operon is dependent on *sigF *and *sigE*, but not on *sigK *genes, in agreement with the current model of the sporulation signal transduction cascade (Table 4). The activation of the sporulation sigma factors is normally coupled to the course of morphogenesis during spore differentiation. It is not clear at this point what mechanism causes the heat-induced short-cut in part of the sporulation program and whether the expressed gene products play an alternative role in heat resistance of the mother cell.

The observed cellular responses suggest that heat stress causes a wide variety of physiological stress. Which type of damage leads to cell death is not clear, but the microarray data indicate that the transcriptional machinery is at least still intact in cells that completely lost the ability to reproduce. This can be concluded from the results that show increasing transcript levels of most genes in the clusters a-j at temperatures from 58–62°C, whereas the majority of the cells died at these temperatures (compare Table 3 and Figure 5).

**Figure 8:**
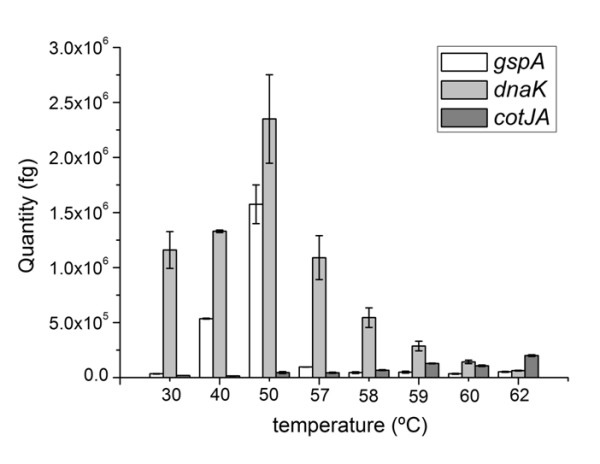
**Transcription level validation with quantitative PCR**. The bars indicate the results of a quantitative PCR (Q-PCR) expressed in equivalents of femto grams (fg) of the *B. subtilis *chromosomal DNA used for calibration (see Methods). The Q-PCR was carried out for 4 primer-probe combinations, designed for the gene encoding *dnaK*, *gspA*, *cotJA *and 23S rRNA genes, as listed in Table 2. The expression levels in femto grams were normalized to the level of 23S rRNA (see the electroferogram in Figure 2).

Expression levels in living and dead cells were further validated by quantitative PCR to assess *gspA*, *dnaK *and *cotJ *transcript levels in *B. subtilis *cells exposed to the heat regime in Figure 1. The results in Table 3 further substantiate the conclusions derived from the microarray results. The transcript levels of *cotJA *keep on increasing over the entire heat regime, which is most likely resulting from transcriptional activity up to 62°C. The SigB controlled *gspA *expression profile occurs as a highly induced, transient response, peaking at 50°C in line with the microarray data. The *dnaK *transcription levels are induced to a smaller extent, but from a much higher reference level at 30°C (Figure 8 and Table 3).

Next, we defined a number of criteria to identify transcripts that could act as biomarkers for cell viability: (i) high expression levels in living bacteria, (ii) relatively low expression levels in heat-inactivated bacteria, and (iii) genes that are widely conserved throughout the bacterial kingdom. We selected a set of 4 genes, all under the control of the HrcA regulator, which matched these criteria; they include *grpE*, *dnaK*, *groES *and *groEL *(see Figure 7). We observed that the above criteria for expression levels of these genes also matched in microarray studies on other bacterial species, where the cause of death was different: *Lactobacillus plantarum *exposed to ultra high-pressure treatments and *Staphylococcus aureus *exposed to biocides (data not shown).

Both *dnak *and *groE *operons are preceded by a SigA-type promoter, which ensures transcription of these genes under growth conditions without stress (in line with the *dnaK *expression levels in table 3), as protein folding is also needed under these conditions. A fascinating question involves the mechanism for the reproducible rapid decrease of these transcripts in bacteria that lost the ability to grow. In living bacteria, the CIRCE element (controlling inverted repeat of chaperone expression), located between the transcriptional and translational start sites of both *dnaK *and *groESL *operons [22], is involved in the regulation of both operons. In the current model the GroE chaperonin, the activator of the HrcA repressor, which binds to CIRCE, is titrated away in the presence of unfolded proteins in an ATP-dependent process, leading to inactivation of HrcA and enhancement of transcription of *dnaK *and *groESL *operons [21]. Thus, the down regulation or degradation of these transcripts in excess of unfolded proteins asks for an extension of this model. No matter what regulatory mechanisms, the identification of 4 transcripts of chaperone genes as biomarker for viability needs to be further validated for a wide range of environmental conditions to determine the lethal conditions for which they are bona fide.

## Conclusion

This study contains a systematic comparison between cultivation-based assessment of cell viability and cultivation-independent indicators for cell viability by sampling and analyzing heat-exposed *B. subtilis *cells going through the transition from culturable to unculturable cells. Accordingly, some viability indicators appear more sensitive than the conventional enumeration of colony forming units (CFU's), while others appear less sensitive measures: during heat stress the extension of lag time for outgrowth, the degradation of 16S rRNA and the induction of classic heat shock genes precede the loss of CFU's, while the loss of membrane permeability and the degradation of 23S rRNA occur after the loss of CFU's. The genome-wide transcriptional response indicates that heat stress causes a wide variety of physiological stresses. Which type or types of cell damage lead to cell death is not clear, but the microarray data indicate that the transcriptional machinery is at least still intact, as a subset of genes is still expressed in cells that lost the ability to reproduce.

The heat-induced transcriptional program includes known heat shock responses as well as the rapid expression of a small number of sporulation and competence genes, the latter only known to be active in the stationary growth phase. Further research needs to assess whether the activation of a small number of genes in these well-studied developmental programs is a result of deregulation due to heat damage or a meaningful, physiological event fitting in the cell's strategy to cope with heat stress. Notably, the transcription of a limited number of genes correlated with cell viability under the applied killing regime. The transcripts of the expressed genes in living bacteria – but silent in dead – include those of essential genes encoding chaperones of the protein folding machinery and may serve as molecular biomarkers for bacterial cell viability.

## Authors' contributions

FHS and RM developed the initial concept for this study. RK, BJK, FHS and RM participated in experimental design and coordination of the study, RK, MPMC and FHS analyzed the data, RK wrote the paper. All authors read, corrected and approved the final manuscript.

## Supplementary Material

Additional file 1 Time-lapse movie of heat-induced expression profiles. The time-lapse movie of expression profiles was constructed by the use of Windows Movie Maker version 5.1 (Microsoft Inc.). All genes were sorted in the gene order present on the *B. subtilis *genome and presented in 32 columns of 96 genes each. Only those genes that contained a detectable transcription level at 30°C were analyzed. This filter led to a total of 75% of the genes (3072 out of 4100 genes). The 29 frames used for the movie consist of 8 measurements from 0–12 minutes and 21 interpolations. The parameters time, temperature and CFU counts were inserted in the microarray images, which were generated with the TIGR MultiExperimentViewer version 4.0.01. The ^2^log ratios were colour-coded, ranging from -3 (green) to +3 (red). Note that there is one region throughout the whole series of profiles with nearly identical expression levels to the reference profile at 30°C. This region contains 67 ORFs from *rplK *to *rpsL *and encodes at least 47 different ribosomal proteins. Click here for file
